# Subject-specific sensitivity of several biomechanical features to fatigue during an exhaustive treadmill run

**DOI:** 10.1038/s41598-024-51296-0

**Published:** 2024-01-10

**Authors:** Christos Chalitsios, Thomas Nikodelis, Georgios Mavrommatis, Iraklis Kollias

**Affiliations:** 1 Biomechanics Laboratory, Department of Physical Education and Sports Sciences, Aristotle University of Thessaloniki, Thessaloniki, Greece; 2 Department of Physical Education and Sports Sciences, Aristotle University of Thessaloniki, Thessaloniki, Greece

**Keywords:** Physiology, Motor control

## Abstract

The aim of the present study was to examine the sensitivity of several movement features during running to exhaustion in a subject-specific setup adopting a cross-sectional design and a machine learning approach. Thirteen recreational runners, that systematically trained and competed, performed an exhaustive running protocol on an instrumented treadmill. Respiratory data were collected to establish the second ventilatory threshold (VT2) in order to obtain a reference point regarding the gradual accumulation of fatigue. A machine learning approach was adopted to analyze kinetic and kinematic data recorded for each participant, using a random forest classifier for the region pre and post the second ventilatory threshold. SHapley Additive exPlanations (SHAP) analysis was used to explain the models’ predictions and to provide insight about the most important variables. The classification accuracy value of the models adopted ranged from 0.853 to 0.962. The most important feature in six out of thirteen participants was the angular range in AP axis of upper trunk C7 (RT_APu_) followed by maximum loading rate (RFD_maxD_) and the angular range in the LT axis of the C7. SHAP dependence plots also showed an increased dispersion of predictions in stages around the second ventilatory threshold which is consistent with feature interactions. These results showed that each runner used the examined features differently to cope with the increase in fatigue and mitigate its effects in order to maintain a proper motor pattern.

Human movement, results from the complex interplay of the nervous, muscular, and skeletal systems, and exhibits unique outcomes due to the adaptability of those systems^1,2^. The interaction of intrinsic-organismic, environmental, and task constraints leads to individualized movement patterns^3^.

Running is a typical example of a fundamental human activity where all the above-described properties can be observed. Running is a demanding activity for each of the three-component structure that controls movement. It becomes even more challenging for the system mechanics when fatigue progressively accumulates. Fatigue and running are two terms that are inextricably linked. Fatigue as a phenomenon has attracted significant research interest over the years and is defined as the decline in various objective measures of performance over a discrete time period^4^.

During exhaustive exercise, central and peripheral fatigue contribute to performance decline^5^. Central fatigue relates to the reduced central nervous system capacity for optimal motor output, while peripheral fatigue involves factors at/or distal to the neuromuscular junction^6–8^. The interplay between fatigue mechanisms and physiological constraints have been reported to influence performance decline^9^.

Researchers have proposed various physiological indices to identify the onset of fatigue accumulation during exercise, including the ventilatory threshold, respiratory compensation point, heart rate deflection point, critical power, physical working capacity at the fatigue threshold, and electromyographic fatigue threshold. These indices are used to estimate physical exertion and mark the metabolic transition from aerobic to anaerobic energy production, where fatigue effects are more likely to be observed^10,11^. Fatigue significantly impacts musculoskeletal mechanics during running, affecting the mechanical behavior of the system. Adverse effects on neuromuscular function can lead to a reduction in mechanical energy transfer during the stretch–shortening cycle^12^ and a decrease in muscle reaction times^13^. Fatigue also influences trunk kinematics, with changes in trunk flexion and extension observed in recreational runners after a fatigue running protocol^14^. Exhaustive running makes the stance phase more variable, complicating athletes' efforts to maintain optimal angular displacements^15,16^. A recent study^17^ highlighted the importance of features like lateral trunk bending and maximum loading rate in predicting the fatigue state of recreational runners.

Since inter-individual variability is a well-established concept^18^, the accumulation of fatigue during running is very probable to cause a unique pattern in the strategy that every runner employs to compensate for the effects of fatigue. Bates et al.^1,19^ identified unique performance characteristics among five elite runners that were masked when a descriptive group approach was adopted. However, running biomechanics are mostly investigated using group-based analyses. While group-based analysis can provide meaningful insights about the differences between groups or/and extract conclusions about universal features that characterize human movement irrespective of the level of expertise^20^, the lack of homogeneity in human movement result in a substantial difficulty in deciphering individual patterns. Especially, using group model statistics, this challenge becomes even harder. On the contrary, subject-specific modeling can be useful in identifying emerging patterns and track changes within a single individual. With the substantial improvement of wearable Inertial Measurement Unit (IMU) sensors in quality, cost and ease of recording reliable data, coaches and runners use such systems to quantify fatigue, training load and running mechanics and explore movement patterns.^21,22^ By taking advantage of the ability these sensors provide to continuously record data (which can lead to an extensive amount of information), and the use of subject-specific modeling, we could obtain more specialized information about the movement changes, and in some cases, outperform group-based models.^23,24^.

Advanced data analysis techniques like supervised machine learning models have been proposed to model complex relationships between biomechanical measures and outcomes of interest^25^. Tree based methods like random forests (RF) have been successfully used in biomechanical research because of their robustness^26,27^. Nevertheless, such tree ensemble algorithms can be more difficult in interpretation despite their potentially satisfactory prediction performance. This drawback can be mediated using model interpretation techniques such as SHAP (SHapley Additive exPlanations) values which can be used for assigning feature importance and interactions among the examined features and thus provide insights about algorithm predictions^28,29^.

Therefore, the purpose of this investigation was to explore through machine learning models (classification) the subject-specific changes in biomechanics of running to exhaustion and map the importance of the measured features, using a physiological threshold as the cut-off criterion. The importance of individuality in the running pattern was supported by Hoitz et al.^30^ which found that kinematic features of running, derived from coronal and transverse plane were the most relevant in providing information on a runner’s unique movement pattern, whereas characteristics of the sagittal plane and ground reaction forces in vertical or anterior–posterior direction were mostly irrelevant.

This study was set to explore the hypothesis that during incremental running to exhaustion some features, which characterize running technique, will have a universal importance amongst the individuals, while others will contribute in a subject-specific way.

## Results

The classification accuracy value range was from 0.853 to 0.962. Classification accuracy and other metrics obtained for each runner are displayed in Table 1. The SHAP based variable importance ranked all the features according to their value for the model, to predict the state above the VT2.

**Table 1:** Classification accuracy, Cohen’s kappa, sensitivity, specificity and F1 score for each runner.

Runner	Accuracy (95% CI)	Kappa	Sensitivity	Specificity	F1-score
R1	0.853 (0.815–0.886)	0.701	0.850	0.857	0.815
R2	0.877 (0.842–0.907)	0.750	0.885	0.867	0.825
R3	0.918 (0.891–0.940)	0.795	0.930	0.885	0.842
R4	0.881 (0.844–0.912)	0.762	0.898	0.863	0.839
R5	0.887 (0.853–0.915)	0.732	0.797	0.927	0.887
R6	0.953 (0.930–0.969)	0.904	0.943	0.961	0.922
R7	0.940 (0.915–0.959)	0.880	0.929	0.952	0.896
R8	0.962 (0.940–0.978)	0.920	0.968	0.953	0.882
R9	0.955 (0.932–0.973	0.902	0.952	0.961	0.854
R10	0.923 (0.895–0.946)	0.827	0.953	0.866	0.792
R11	0.927 (0.901–0.948)	0.821	0.972	0.823	0.724
R12	0.952 (0.929–0.970)	0.902	0.952	0.953	0.881
R13	0.948 (0.923–0.967)	0.895	0.973	0.921	0.851

**Figure 1:**
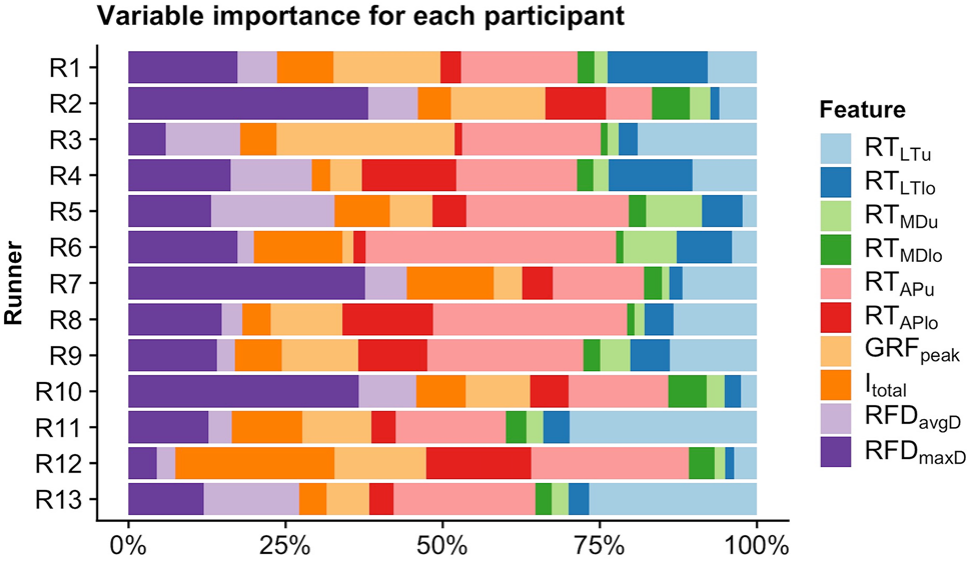
SHAP based variable importance for each participant.

In Fig. 1, the proportion of feature importance for each runner is presented. Τhe feature RT_APu_ appeared to be the most important for prediction in 6 out of 13 participants. Also, RFD_maxD_ appeared first in 3 out of 13 and RT_LTu_ in 2 cases respectively. GRF_peak_ and I_total_ were the most important features for 1 out of 13 of the sample participants.

**Figure 2:**
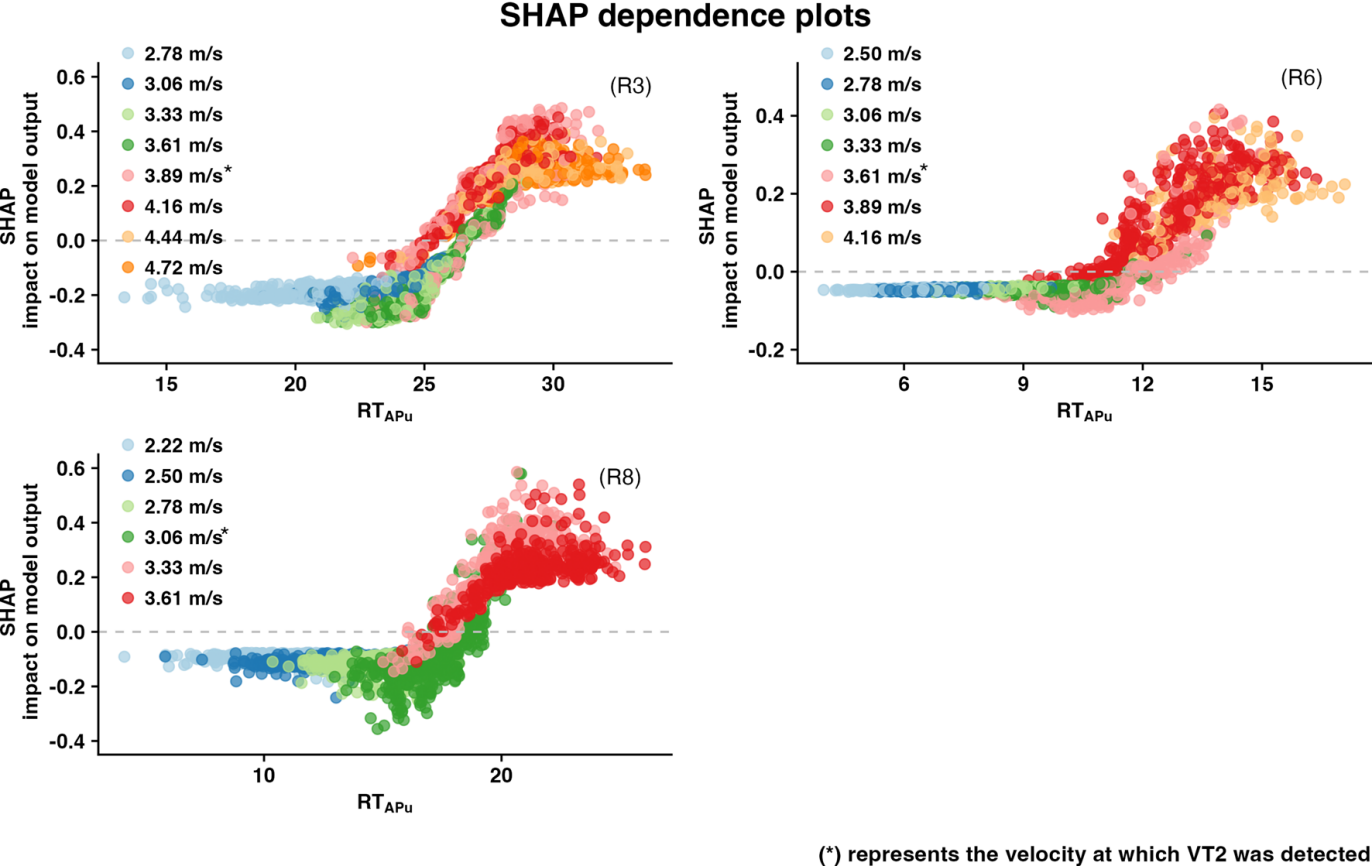
SHAP dependence plots for the three representative participants with RT_APu_ (lateral trunk flexion/extension) as their most influential feature. SHAP dependence plots show the contribution of a feature to the model based on the feature’s distribution. In this plot each point shows an observation from the individual datasets, the X-axis line shows the value of the feature in that instance, and the Y-axis shows the SHAP value for that feature (RT_APu_) that indicates the effect of that feature with that specific value on the prediction. The unit of X-axis is the same as the unit of the feature (for RT_APu_—degrees), and the Y-axis is the SHAP value for predicting pre or post the VT2. Color coding has been used to show the velocity stages of the observations. SHAP values above the *y* = *0* lead prediction values towards the target class (after VT2) and the opposite is true for SHAP values less than *y* = *0*.

Figure 2 displays the SHAP dependence plot which describes feature effects on the predictions. The *y-axis* represents the SHAP value and *x-axis* the raw feature values and so the plot shows the features’ overall influence on the model predictions and thus provides an intuitive way to understand the fitted model.

## Discussion

The aim of this study was to classify changes in subject-specific running to exhaustion patterns based on the VT2 using RF classifiers. The results of the current study supported the first part of the initial hypotheses and demonstrated that using an RF approach, robust and accurate classification could be achieved (Ahamed et al., 2019).

While each participant had a different mix of important predictor variables, RT_APu_ was the most or second most important feature in the majority of the participants. This is in line with previous findings from group-based analysis and also verifies the initial hypothesis of the study^17^. Winter et al.^2^ identified the critical role of trunk frontal plane kinematics (RT_APu_) for balance control due to the trajectory of the center of mass which is medial to the base of support. Also, lateral flexion (RT_APu_) of the upper trunk towards the supporting leg serves as an assisting mechanism to oppose the abduction torque mainly induced by GRF during running^31^. Therefore, regulation of this feature is important for an effective running pattern.

Nevertheless, although the majority of runners exhibit a behavior that was characterized from RT_APu_ there were also individuals who did not followed that pattern and had a totally different set of features describing their performance. For example, subjects 2 and 7 displayed a very specific behavior regarding feature importance, which at the same time differed from all other participants (Fig. 1). Other researchers that studied the walking and running gait patterns in healthy individuals also reported the presence of distinct patterns^32^. Putting fatigue in the equation, it is reasonable to expect that individuals will respond with a specific motor solution seeking a more energy-efficient movement pattern based on the constraints imposed from their anatomy, morphology, physiology and level of training. Apart from the energy efficiency hypothesis^33^, fatigue in this case could be the control variable, the scaling of which may be responsible for moving to a different attractor^34^ according to the dynamic systems theory perspective. This theory posits that the behavioral state (attractor) of an order parameter (running mechanics in the present case) is dependent on the scaling of a control parameter (duration or speed of running). Such transition phases are characterized by highly variable patterns^35^ of the order parameter.

Therefore, the expected motor response to fatigue could be different not only for the magnitude level of a specific feature, but also for all selected features that are triggered as a response to the imposed demands. The SHAP dependence plots for the most influential feature are also pointing out the specificity of the response, with some individuals exhibiting more consistent patterns, while others have more variance in their predictions. Specifically, it is observed that in all runners, for the first stages of the test, where velocity is at the low end of their respected continuum to exhaustion, predictions are very consistent with a negative prediction impact on the overall model. That is not the case as running velocities increase and especially in the transition phase (between 55—75% of the total test duration in the current study) where variability of prediction is evident. At this point it is crucial to report that increasing velocities without the presence of fatigue does not influence the variability of the response. This finding is valid up to velocities up to 100% of maximal aerobic velocity^36^ which corresponded to velocities up to ~ 5.5 m/s. The final velocities in our study ranged from 3.89 to 5 m/s. Therefore, the dispersion of prediction values, originating from the intermediate stages of velocity, points out the existence of a certain degree of variability in the movement response pattern during these stages, where fatigue apparently kicks-in. For example, in runners 6 and 8 (Fig. 2) there is a notable consistency in predictions with increasing velocity but this is not the case for runner 3 (Fig. 2) where significant variability is present.

Also, the vertical dispersion (when for about the same area of the feature’s distribution the impact on overall model may be very different) in the SHAP values that appears in some individuals for fixed feature values, may be related to an interaction effect with other features^29^. This highlights the fact that an instance of SHAP values for a feature is not solely dependent on the value of that feature, but is also influenced from the values of other features at that instance. In Fig. 2 it is observed that at the early stages of the test, where everybody runs in a comfortable velocity, there is limited vertical dispersion in the SHAP values, relatively to later stages of the test. Possibly this phenomenon suggests that the model can capture the compensations, or interconnections of available features, that occur while the body tries to adapt and maintain proper technique.

The importance of these two characteristics of the SHAP plots, as interpreted above, is that they account for feature interaction and the variability of prediction around the transition phase. This could provide a direction regarding what velocities are those in which training should take place to make the movement pattern more stable and mitigate the effects of fatigue at higher intensities while maintaining the proper technique^15,37,38^. As stated previously observing Fig. 2 can reveal that there are individuals where predictions are consistent and in line with the natural succession of velocities as going towards the end of the test and makes clear for example that runners 6 and 8 exhibit a stable pattern up to 3.61 m/s. Beyond those velocities significant variation and interactions are present which likely suggest alterations in the running biomechanical pattern.

A substantial body of the literature has examined mostly single and discrete variables (e.g. stride time, cadence) often in isolation from other features, resulting in conflicting outcomes about the effects of fatigue during running. For example, measuring contact time after fatigue in a study by Morin et al. (2011a) in ultramarathon runners who performed a 24-h treadmill test showed a decrease. However, it has to be clear that fatigue during an incremental to exhaustion test and ultra marathons is regulated by very different mechanisms. Moreover, there are reports of reductions in peak knee flexion angle during stance^40^, whereas others reported an increase^41^. The differentiation of the protocols in terms of test velocity (constant—variable) may also have contributed to the observed difference. Nevertheless, this type of univariate statistical approach may also be less sensitive in detecting changes in overall running patterns^42^. In contrast, the current study supports previous research which suggests that examining multivariate and/or multi-segment changes may better quantify the overall biomechanical changes that occur during running (Phinyomark et al., 2015). It should be noted that although data points from each step in the entire trial were recorded for building the subject-specific models, the sample was relatively small to account for the substantial population variations. Other variables that often have shown to be sensitive to fatigue such as flight time, contact time, vertical stiffness, etc., were not measured in this study and therefore this could impact the model output.

A critical point concerns the calculation of the RFD feature for which the time component is imperative. The validity of the treadmill force plate set up was checked for its structural stiffness over several loads with dead weights with the motor on and off and was found quite higher than leg stiffness during running^45^. Yet any amount of dampening or delay in the force transmission through the treadmill to the force plates may affect the accuracy of RFD value or even GRF_peak_ by smoothening them due to “filtering” of high frequencies. Nevertheless, dampening occurs when running on track, grass and practically in several natural running surface that runners tend to train. Another potential limitation could arise from the a) the timing variability of VT2 detection and b) the individuality of the physiological or psychological response. Since there is a certain degree of subjectivity in the identification process and interpretation criteria in the literature this could bring some uncertainty in the precise estimation of VT2, and also the perception of fatigue could differ due to individual physiological or psychological differences. While this method offers an interesting angle for exploration, we recognize the inherent difficulty in pinpointing a precise crossing point at which fatigue becomes conspicuous and emerges. The intricate and multifaceted nature of fatigue, along with its manifestation in kinematic patterns, contributes to this challenge. Nevertheless, the accuracy of the models supports the use of VT2 as a criterion for supervised machine learning approach. Future research should focus on examining alternative approaches for detecting the onset of fatigue accumulation and further understanding its impact on kinematic variations.

It should be reported that the present research setup and methodology points out associations between selected features but no cause-and-effect relationships. However, the SHAP values, can assist to gain a deeper understanding of the complex relationships between the features and the output, as well as the interactions among the features themselves. Although these associations are not entirely interpretable, they form an inspiring challenge to study the complexity of the system as a whole. For example, this can be pursuit following a dynamic systems perspective (how musculoskeletal and biological constraints interact with the task).

## Materials and methods

### Participants

Thirteen (13) male recreational runners (age = 37.84 ± 4.53 years, height = 178.15 ± 5.37 cm, weight = 78.85 ± 6.89 kg) voluntarily participated in the study. All runners were healthy and free of any neuromuscular or musculoskeletal disorders. All participants had to have at least three years of systematic training and racing at distances greater than 10 km. The range of performances across the participants in the study was between 49 and 57 min for 10 km races. The study protocol was approved by the Institutional Research Review Board (Aristotle University Research Ethics and Bioethics Committee: ΕΗ-12/2020) and was conducted in accordance with the Declaration of Helsinki. Written informed consents were obtained from all participants.

### Protocol and instrumentation

Both biomechanical and physiological data were collected during an incremental running to exhaustion test on a treadmill (Impulse RT700, UK). All tests took place between 14.00 and 18.00 pm. Familiarization with the measuring equipment was pursued with participants a day before the trial when they came to the lab for an easy five-minute run, wearing all the measuring equipment without logging any data. On the testing day the participants were asked not to eat anything at least 3 h prior to testing. At this day the procedure included an eight-minute light warmup at a self-comfort velocity followed by 5 min of dynamic stretching. The main running task were initiated with participants running at a velocity equivalent to 85% of their 10 k tempo (most recent race pace) which was approximately between 2.5 and 3.61 m/s. All subjects performed 3-min stages with a steady increase on the workload of 0.28 m/s until they were unable to continue and voluntarily interrupted the test. To ensure that participants reached significant levels of exhaustion the following criteria were used: Respiratory Exchange Ratio ≥ 1.1, Heart Rate ≥  ± 10 beats × min^−1^ age predicted HRmax, and RPE > 17. Respiratory data ($\dot{V}$O_2_ and $\dot{V}$CO_2_) were recorded through a portable gas analyzer (PNOE, ENDO Medical, Palo Alto, CA). Ground reaction force data obtained from a dual force plate system (k-Delta, K-Invent, Biomechanique, Montpellier) with a sampling frequency of 516 Hz on which the treadmill was securely mounted. Also, torso kinematics were quantified using a pair of USB connected 6 DoF IMU_s_ (k-sens, K-Invent, Biomechanique, Montpellier) with 218 Hz sampling frequency. From angular velocity data, the angular displacement was calculated through integration of angular velocity around the axis of the interest. Systematic drift of the gyroscopes that appeared during integration was removed using least squares regression methods. The minimum detectable step (resolution) for the IMU sensors is 4 mg/LSB (Least Significant Bit) for the accelerometer, and 0.06°/s for the gyroscope. The maximum detectable value for the accelerometer was ± 16 g and for gyroscope ± 2000°/s. The sensors were fixed on C7 and L5 respectively. Both systems were internally synchronized.

### Data analysis

#### Pre-processing

Raw kinetic and kinematic signals were filtered with a second order Butterworth filter with a cutoff frequency at 30 and 15 Hz respectively. All the features that were extracted from the ground reaction force and the IMU data, represented discrete values from every step (Table 2). For each footfall, contact times were determined with backward and forward search of the point when the curve gradient was equal to zero and setting a threshold for the vertical force signal exceeded 40 N.

**Table 2:** Extracted features from the raw signals.

Abbreviation	Feature	Unit
GRF_peak_	Max force value per contact	N·kg^−1^
RT_LTu,_ RT_MDu,_ RT_APu_	Angular range around the three axes of motion (C7) RT_LTu_ = vertical rot, RT_MDu_ = flex/ext, RT_APu_ = lateral flexion	deg°
I_total_	Total Impulse	N·s
RT_LTlo,_ RT_MDlo,_ RT_APlo_	Angular range around the three axes of motion (L5) RT_LTlo_ = vertical rot, RT_MDlo_ = flex/ext, RT_APlo_ = lateral flexion	deg°
RFD_maxD_	Maximum value of rate of force development until GRF_peak_	kN·s^−1^
RFD_avgD_	Average value of rate of force development until GRF_peak_	kN·s^−1^

A moving average filter of eleven breath window was adopted to smooth ventilation data. For the second ventilatory threshold (VT2) identification, two experienced independent researchers were asked to examine $\dot{V}$E/$\dot{V}$O_2_—$\dot{V}$E/$\dot{V}$CO_2_ curves and report the specific time point. In the literature, the VT2 which is closely related to anaerobic processes, is defined as an over proportional increase in $\dot{V}$E vs $\dot{V}$O_2_ output^46^. The average estimation was defined as the time point of data division in two conditions: before and after VT2. A twenty second data window before and after that point was removed for every individual. The datasets were searched for outliers according to a rule of ± 3 standard deviations. If a point were ± 3 standard deviations away from the local mean value (3’ stage), that point’s value was replaced with nearest non outlier point value. In any case in all of the thirteen datasets the percentage of outliers were less than 1%. Finally, thirteen *n* **x** m matrices were created, one for every participant with *n* representing rows (observations) and *m* columns (features) with *n* ranging between 2347 and 3577 among participants.

#### Subject specific models

Random forests (RF) were used for classification of the pre and post conditions. RF implementation was performed as originally described by Breiman^47^. The algorithm combines many decision trees grown in random subsets selected with bootstrap aggregation from the feature space. Also, each time a split is made a random sample of the available features are considered. Since predictions are produced from the majority vote (average prediction of individual trees) of the produced trees, the described procedure has the advantage to decorrelate the trees and provide good results in terms of accuracy and highlighting the importance of the supplied features. Model results validation was checked by randomly splitting the data into training (80%) and testing (20%) sets. The algorithm was trained with tenfold cross validation in the training set and tested the predictions in the remaining 20% of the data. This procedure was performed separately for every individual and the related performance metrics for each runner appear in Table 1.

In classification problems, large differences in proportions of the response (dependent) variable may have a significant negative impact on model fitting. For that reason, special consideration was taken regarding representative proportions of classes in the response variable during splitting the datasets because of the inter-individual variability in the time points where the VT2 appeared. If large disparity between classes was identified, subsampling with random under-sampling was adopted. Random under-sampling seeks to randomly select and remove instances from the majority class and so to provide a balanced proportion of the classes consisting the response.

RF is sensitive to parameters such as number of trees that will be grown and number of predictors to be considered. For this reason, grid search for optimal values of those parameters was adopted, using out-of-bag error minimization as criterion selection. For feature selection a recursive elimination process was adopted based on accuracy values. This iterative approach aimed to identify the subset of features that have the greatest impact on the accuracy of the model. ﻿It should also be noted that to ensure the method was entirely subject-specific, parameter tuning was conducted separately within each individual. Accuracy (the number of correctly predicted data points out of all the data points), sensitivity (the true positive recognition rate), specificity (the proportion of actual negatives, which got predicted as the negatives) and Cohen’s Kappa coefficient (a measure of how closely the instances classified by the machine learning classifier matched the data labeled as ground truth, controlling for the accuracy of a random classifier as measured by the expected accuracy) were used to assess model’s performance (Table 1). All models were built in Python 3.6 with scikit-learn 1.0.1. Finally, SHAP (SHapley Additive Explanations) with TreeSHAP implementation^29^ were used as proposed by Lundberg & Lee^28^ to interpret how the adopted RF models yielded their predictions. This methodological approach provides consistent and accurate attribution values for each feature within each prediction model^29^. SHAP can provide a visualization of the overall feature importance for all the individual models that were fitted to understand the different combinations of relative importance needed for each of the participant’s predictions. Also, SHAP dependence plots examined how a feature’s attributed importance changes as its value varies within its distribution range^29^. That is a model agnostic, unified approach for explaining the outcome of any machine learning model. SHAP values evaluate the importance of the output resulting from the inclusion of feature A for all combinations of features other than A. SHAP analysis was carried out using the SHAP python module^48^. Since in the current study a subject-specific design and analysis was adopted, we did not include any null hypothesis significance testing (NHST).

## Conclusions

In the present study, each individual exhibited different changes in overall running mechanical parameters in predicting the post VT2 state. These findings also support the efficacy of machine learning modeling approach for understanding the complexities of running gait patterns based on collecting large amount of data in a laboratory setting. Overall, the results of this study showed that the production of work in a fatigued condition results in subject-specific changes in biomechanical running patterns, possibly in an effort to compensate and optimize the system function to account for the imposed demands based on individual movement characteristics or constraints.

## Data Availability

The datasets generated and/or analyzed in the current study are available from the corresponding author upon reasonable request.

## References

[1] Bates BT (1996). Single-subject methodology: An alternative approach. Med. Sci. Sports Exerc..

[2] Winter DA (1995). Human balance and posture control during standing and walking. Gait Posture.

[3] Button C, Seifert L, Chow JY, Davids K, Araujo D (2020). Dynamics of Skill Acquisition: An Ecological Dynamics Approach.

[4] Enoka RM, Duchateau J (2016). Translating fatigue to human performance. Med. Sci. Sports Exerc..

[5] Gandevia SC (2001). Spinal and supraspinal factors in human muscle fatigue. Physiol. Rev..

[6] Taylor JL, Amann M, Duchateau J, Meeusen R, Rice CL (2016). Neural contributions to muscle fatigue: From the brain to the muscle and back again. Med. Sci. Sports Exerc..

[7] Allen DG, Lamb GD, Westerblad H (2008). Skeletal muscle fatigue: Cellular mechanisms. Physiol. Rev..

[8] Fitts RH (1994). Cellular mechanisms of muscle fatigue. Physiol. Rev..

[9] Millet GY, Lepers R (2004). Alterations of neuromuscular function after prolonged running, cycling and skiing exercises. Sports Med.

[10] Bergstrom, H. C. *et al.* The relationships among critical power determined from a 3-min all-out test, respiratory compensation point, gas exchange threshold, and ventilatory threshold. 10.1080/02701367.2013.784723 10.1080/02701367.2013.784723 23930549

[11] Wasserman K, Whipp BJ, Koyl SN, Beaver WL (1973). Anaerobic threshold and respiratory gas exchange during exercise. J. Appl. Physiol..

[12] Mizrahi J, Verbitsky O, Isakov E (2000). Fatigue-related loading imbalance on the shank in running: A possible factor in stress fractures. Ann. Biomed. Eng..

[13] Mizrahi J, Verbitsky O, Isakov E (2001). Fatigue-induced changes in decline running. Clin. Biomech..

[14] Koblbauer IF, van Schooten KS, Verhagen EA, van Dieën JH (2014). Kinematic changes during running-induced fatigue and relations with core endurance in novice runners. J. Sci. Med. Sport.

[15] Chen TLW (2020). Changes in segment coordination variability and the impacts of the lower limb across running mileages in half marathons: Implications for running injuries. J. Sport Health Sci..

[16] García-Pinillos F (2020). Does fatigue alter step characteristics and stiffness during running?. Gait Posture.

[17] Chalitsios C, Nikodelis T, Konstantakos V, Kollias I (2022). Sensitivity of movement features to fatigue during an exhaustive treadmill run. Eur. J. Sport Sci..

[18] Hashizume S, Hobara H, Kobayashi Y, Tada M, Mochimaru M (2018). Inter-individual variability in the joint negative work during running. Sports Med. Int. Open.

[19] Bates BT, Osternig LR, Maso BR (1979). Variations in velocity within the support phase of running.

[20] Lubetzky-Vilnai A, Ciol M, McCoy SW (2014). Statistical analysis of clinical prediction rules for rehabilitation interventions: Current state of the literature. Arch. Phys. Med. Rehabil..

[21] Willy RW (2018). Innovations and pitfalls in the use of wearable devices in the prevention and rehabilitation of running related injuries. Phys. Ther. Sport.

[22] Clermont CA, Benson LC, Osis ST, Kobsar D, Ferber R (2019). Running patterns for male and female competitive and recreational runners based on accelerometer data. J. Sports Sci..

[23] Rodríguez-Martín D (2017). Home detection of freezing of gait using support vector machines through a single waist-worn triaxial accelerometer. PLoS One.

[24] Benson LC, Clermont CA, Bošnjak E, Ferber R (2018). The use of wearable devices for walking and running gait analysis outside of the lab: A systematic review. Gait Posture.

[25] Halilaj E (2018). Machine learning in human movement biomechanics: Best practices, common pitfalls, and new opportunities. J. Biomech..

[26] Ahamed NU (2019). Subject-specific and group-based running pattern classification using a single wearable sensor. J. Biomech..

[27] Ahamed NU (2018). Using wearable sensors to classify subject-specific running biomechanical gait patterns based on changes in environmental weather conditions. PLoS One.

[28] Lundberg SM, Lee SI (2017). A unified approach to interpreting model predictions. Adv. Neural Inf. Process. Syst..

[29] Lundberg, S. M., Erion, G. G. & Lee, S.-I. Consistent individualized feature attribution for tree ensembles. (2018).

[30] Hoitz F, von Tscharner V, Baltich J, Nigg BM (2021). Individuality decoded by running patterns: Movement characteristics that determine the uniqueness of human running. PLoS One.

[31] Kulmala J-P (2017). Whole body frontal plane mechanics across walking, running, and sprinting in young and older adults. Scand. J. Med. Sci. Sports.

[32] Phinyomark A, Osis S, Hettinga BOS, Ferber R (2015). Kinematic gait patterns in healthy runners: A hierarchical cluster analysis. J. Biomech..

[33] Selinger JC (2022). Running in the wild: Energetics explain ecological running speeds. Curr. Biol..

[34] Kelso JAS (1995). Dynamic Patterns The Self-Organization of Brain and Behavior.

[35] Granatosky MC (2018). Inter-stride variability triggers gait transitions in mammals and birds. Proc. R. Soc. B: Biol. Sci..

[36] Belli A, Lacour JR, Komi PV, Candau R, Denis C (1995). Mechanical step variability during treadmill running. Eur. J. Appl. Physiol. Occup. Physiol..

[37] García-Pinillos F (2020). How do amateur endurance runners alter spatiotemporal parameters and step variability as running velocity increases? A sex comparison. J. Hum. Kinet..

[38] Derrick TR, Dereu D, McLean SP (2002). Impacts and kinematic adjustments during an exhaustive run. Med. Sci. Sports Exerc..

[39] Morin JB, Samozino P, Millet GY (2011). Changes in running kinematics, kinetics, and spring-mass behavior over a 24-h run. Med. Sci. Sports Exerc..

[40] Chan-Roper M, Hunter I, Myrer JW, Eggett DL, Seeley MK (2012). Kinematic changes during a marathon for fast and slow runners. J. Sports Sci. Med..

[41] Jewell C, Boyer KA, Hamill J (2017). Do footfall patterns in forefoot runners change over an exhaustive run?. J. Sports Sci..

[42] Meardon SA, Hamill J, Derrick TR (2011). Running injury and stride time variability over a prolonged run. Gait Posture.

[43] Marotta L, Buurke JH, van Beijnum BJF, Reenalda J (2021). Towards machine learning-based detection of running-induced fatigue in real-world scenarios: Evaluation of IMU sensor configurations to reduce intrusiveness. Sensors.

[44] Phinyomark A, Hettinga BA, Osis S, Ferber R (2015). Do intermediate- and higher-order principal components contain useful information to detect subtle changes in lower extremity biomechanics during running?. Hum. Mov. Sci..

[45] McMahon TA, Greene PR (1979). The influence of track compliance on running. J. Biomech..

[46] Meyer T, Lucía A, Earnest CP, Kindermann W (2005). A conceptual framework for performance diagnosis and training prescription from submaximal gas exchange parameters: Theory and application. Int. J. Sports Med..

[47] Breiman L (2001). Random forests. Mach. Learn..

[48] Lundberg SM (2020). From local explanations to global understanding with explainable AI for trees. Nat. Mach. Intell..

